# Climate justice in higher education: a proposed paradigm shift towards a transformative role for colleges and universities

**DOI:** 10.1007/s10584-023-03486-4

**Published:** 2023-02-09

**Authors:** Alaina Kinol, Elijah Miller, Hannah Axtell, Ilana Hirschfeld, Sophie Leggett, Yutong Si, Jennie C. Stephens

**Affiliations:** 1 Northeastern University School of Public Policy and Urban Affairs, Boston, MA USA; 2 Northeastern University College of Science, Boston, MA USA

**Keywords:** Climate policy, Climate justice, Higher Education, Green New Deal, Energy democracy, Climate transformation

## Abstract

Moving beyond technocratic approaches to climate action, climate justice articulates a paradigm shift in how organizations think about their response to the climate crisis. This paper makes a conceptual contribution by exploring the potential of this paradigm shift in higher education. Through a commitment to advancing transformative climate justice, colleges and universities around the world could realign and redefine their priorities in teaching, research, and community engagement to shape a more just, stable, and healthy future. As inequitable climate vulnerabilities increase, higher education has multiple emerging opportunities to resist, reverse, and repair climate injustices and related socioeconomic and health disparities. Rather than continuing to perpetuate the concentration of wealth and power by promoting climate isolationism’s narrow focus on technological innovation and by prioritizing the financial success of alumni and the institution, colleges and universities have an opportunity to leverage their unique role as powerful anchor institutions to demonstrate climate justice innovations and catalyze social change toward a more equitable, renewable-based future. This paper explores how higher education can advance societal transformation toward climate justice, by teaching climate engagement, supporting impactful justice-centered research, embracing non-extractive hiring and purchasing practices, and integrating community-engaged climate justice innovations across campus operations. Two climate justice frameworks, Green New Deal-type policies and energy democracy, provide structure for reviewing a breadth of proposed transformational climate justice initiatives in higher education.

## Introduction

Higher education leaders often claim that their institutions, through research and teaching, contribute to addressing humanities biggest challenges (Gamoran 2018; Stewart and Valian 2018). In practice, however, it is unclear to what extent higher education is contributing to the transformative changes that society needs to confront the intersecting crises of worsening climate change, growing economic injustice, and increasing health disparities. In this new era of cascading and intersecting catastrophes, a cumulative polycrisis (Tooze 2022), new opportunities, and responsibilities are emerging for the role of higher education in society (Homer-Dixon and Rockström 2022). Globally, the higher education sector is being challenged to respond and innovate to contribute more to climate justice and global sustainability (Steele and Rickards 2022; UNESCO 2022), yet as the climate crisis intensifies, it is increasingly clear that higher education’s commitment to climate justice is insufficient (UNESCO 2022). When the climate action plans and policies of universities focus on technology-based mitigation which, like the climate action plans of so many other organizations and jurisdictions, fail to consider equity and systemic change, climate injustices are exacerbated. With the continued concentration of wealth and power among individuals and organizations who are already privileged, public support for higher education is being reduced, and the priorities of higher education institutions are increasingly aligned with the priorities of the rich and powerful rather than focusing on the public good. Unfortunately, most of the richest individuals and organizations with strong affiliations with higher education institutions are resisting, rather than investing in, a transformative response to the climate crisis and other intersecting crises (Kenner 2019; Stephens 2020). With growing recognition that the climate crisis is a symptom of larger socioeconomic and political dysfunction, a climate justice approach embraces a transformative lens focused on social and financial innovation, which is a paradigm shift from the more mainstream technocratic way of conceptualizing climate “solutions” (Sultana 2022).

The urgent need for societal transformation to simultaneously address worsening economic and health inequities and growing climate vulnerabilities has become more obvious since the wealthiest billionaires in the world more than doubled their wealth since the beginning of the pandemic (Ahmed et al. 2022). Acknowledging the injustice of these unsustainable trends, higher education has the potential and opportunity to leverage substantial intellectual, financial, physical, and labor resources to advance transformation and reduce devastating human suffering from economic and climate injustices. Through diverse mechanisms, the higher education sector has multiple opportunities to explore, support, and advance transformative social change toward a more just and stable future. Most universities, however, are not yet wielding their influence and impact to encourage and prioritize social innovations and social change for climate justice (Kelly et al. 2022a; Steele and Rickards 2022). Unfortunately, many university practices discourage and devalue social innovations toward climate justice (Patel 2021).

Moving beyond technocratic approaches to climate action, climate justice articulates a paradigm shift in how organizations think about their response to the climate crisis. Climate justice broadens responsibility to redress the legacy of injustice and exploitation resulting from systemic practices, policies, and priorities that perpetuate inequities in climate vulnerabilities—locally, regionally, and globally (Sultana 2022; Stephens 2020; Yeampierre 2020). Advancing climate justice, therefore, necessitates not only directing resources away from fossil fuels and carbon emissions, but also actively engaging with systemic changes that disrupt the structures that have emboldened corporate influence and decades of climate obstruction of climate policy. Grassroots behavior changes alone are insufficient to address climate injustices, and are definitely an inadequate approach to minimizing the climate impacts of higher education (Eichhorn et al. 2022). To facilitate and support systemic changes, colleges and universities have an opportunity to reimagine their internal and external initiatives to prioritize direct engagement and investment in transformative social change toward more just systems locally, regionally, nationally, and internationally (Rempel and Gupta 2022).

As a concerned, collaborative group of graduate student researchers, undergraduate students, and a professor, we draw on our collective knowledge and experiences to offer a novel framework to considering how higher education can contribute to societal transformation for climate justice. In this paper, we make a conceptual contribution by exploring the potential of a paradigm shift in higher education; through a commitment to advancing transformative climate justice, colleges and universities around the world could realign and redefine their priorities in teaching, research, and community engagement to shape a more just, stable, and healthy future. Our methodological approach includes (1) a synthesis of our collective empirical knowledge and experiences engaging with and reviewing climate action planning processes at multiple colleges and universities, and (2) application of two climate justice frameworks, the Green New Deal and energy democracy, to explore how higher education can advance and accelerate a transformative climate justice response to humanity’s intersecting crises. This paper is primarily a conceptual contribution because despite our engaged analysis of university sustainability initiatives and despite focused efforts to systematically review current university climate commitments, we have not been able to identify empirical examples to demonstrate the novel paradigm shift that we are proposing. To complement our conceptual proposal, we have reviewed and synthesized a breadth of specific actionable initiatives aligned with this paradigm shift that we present within two compatible climate justice frameworks.

This paper first reviews climate justice literature to explain how a commitment to climate justice is a paradigm shift in higher education. Then, to explore the transformative potential of specific higher education climate justice initiatives, we apply two climate justice frameworks to our empirical assessment of innovative opportunities in higher education. We end with conclusions and recommendations.

## Higher education and climate justice

More frequent and extreme disruptive climatic events are adversely impacting water access, food production, physical and mental health, and physical and economic infrastructure—particularly for vulnerable communities around the world (IPCC 2022). In this new era of multiple intersecting globally connected crises (Tooze 2022), it is more critical than ever before that higher educations’ commitments to climate action are directly linked to social justice and economic justice (Chankseliani and McCowan 2021; Harlan et al. 2015). Because of the inequities of climate impacts, i.e. the devastation of the climate crisis is being experienced very differently among more vulnerable people, communities, and places, transformative climate action requires conceptually and empirically linking the climate crisis with social injustices, economic inequities, and health disparities (Cappelli et al. 2021; Singer 2018). The interconnected impacts of climate disasters, air and water pollution, food scarcity, substandard healthcare, and other climate-related hazards plague lower-income, under-invested-in communities disproportionately (Angelsen and Dokken 2018; World Health Organization 2021). Furthermore, the inequities of environmental vulnerabilities including racial and gender disparities are a symptom of systemic injustices linked to the history of racial injustice in policies and investments within established governing bodies and decision-making processes (Bullard and Johnson 2000; Roberts-Gregory 2021). The unequal distribution of harm is not an accident and is not isolated to one country or region; in many places, including the USA (Bullard and Johnson 2000), industrial facilities for fossil fuel extraction, refining, and distribution have been intentionally relegated to poorer communities and communities of color by policymakers at all levels of government (McLeod 2007). Around the world, disadvantaged communities with less access to political power, resources, and information have less capacity to mobilize against the allocation of disproportionate environmental burdens (Banzhaf et al. 2019).

Disparities in current and predicted climate impacts exist at multiple levels, including global inequities, within-country inequalities, and regional inequities, particularly across differences in race, class, gender, and disability (Frosch et al. 2018). These relationships are characterized by reinforcing vicious cycles: initial inequality causes the disadvantaged groups to suffer disproportionately from the adverse effects of climate change, resulting in greater subsequent inequalities (Sultana 2022; Islam and Winkel 2017). The global injustice of those countries that have contributed the least to the climate crisis are among the most vulnerable is now widely acknowledged (Watts et al. 2021). It is unjust that countries with virtually no contribution to the climate crisis are more quickly and intensely falling victim to temperature rise, increasingly unpredictable weather disrupting livelihoods and food production, limited resources exacerbating armed conflict, and forced climate migration caused by the consumption and pollution of industrialized nations (United Nations Meetings Coverages and Press Releases 2019). Many of these inequitable exposures and vulnerabilities directly result from colonial legacies of violently coopting environments and resources from communities for unsustainable extraction (Howitt 2020). Given the pervasiveness of these injustices, a transformative climate justice lens is imperative for effective climate action (Sultana 2022).

### A paradigm shift: moving beyond climate isolationism toward climate justice in higher education

**Fig. 1:**
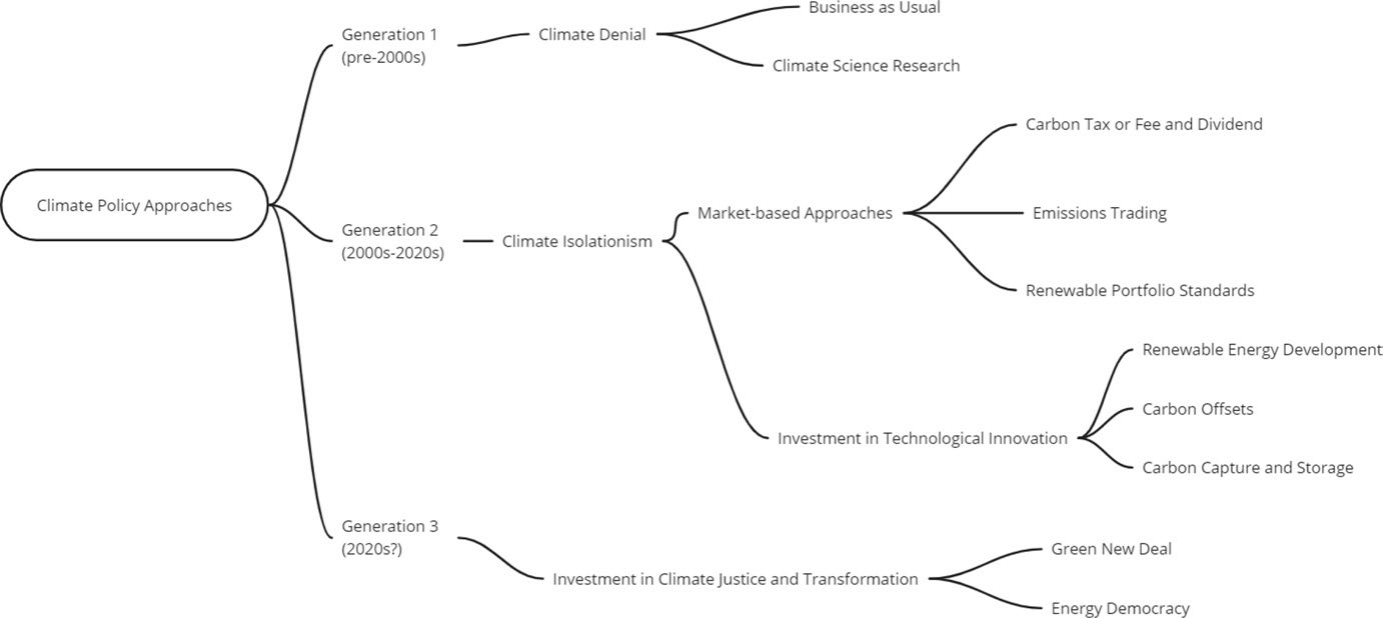
Institutional approaches to climate policy. Three generations of climate policies have evolved over time. The generations are additive, in that climate denial and climate isolationism remain the most common approaches to climate policy into the 2020s. What distinguishes the third generation is that it features investment in addressing the underlying social drivers of climate change and vulnerabilities thereto

The inadequacy of humanity’s response to the climate crisis over the past 30 years can be attributed, at least in part, to climate isolationism, the common framing of climate change as an isolated, discrete, scientific problem in need of economic and technological solutions (Fig. 1) (Stephens 2020, 2022a). In the first generation, before the 2000s, climate policy was dominated by denial and investment in climate science research to better understand the problem. In the second generation, between 2000 and 2018, narrow technocratic approaches dominated including market-based approaches and technological innovation. The third generation expands to include calls for transformative public investment in climate justice. Large-scale investments in climate justice can be considered a third generation of climate policy, following the early era of denying or largely ignoring climate change and recent market and technology-based efforts, which featured some more effective approaches (renewable energy development and standards) than others (carbon capture and offsets).

Non-transformative approaches to climate policy extend beyond governmental policy and are also widespread in institutional decision-making. Policymakers and organizations leaders working on climate mitigation and adaptation within a lens of climate isolationism often focus on quantitative greenhouse gas emission reductions and temperature change while inadvertently ignoring the societal complexities and potential social innovations associated with goals defined by these quantitative measures. Climate isolationism diminishes the potential for transformative social change by understating the relevance of investing in social innovation, social infrastructure, and social justice (Anderson and Peters 2016). When the climate crisis is framed as a scientific problem in need of a technological fix, public discourse and imagination on changing the underlying societal and economic structures is constrained. Climate isolationism is a form of climate obstruction (Ekberg et al. 2022; Lamb et al. 2020) because it delays climate action by obfuscating links between the worsening climate crisis and the concentration of wealth and power among those profiting from maintaining fossil fuel reliance who are resisting transformative change (Stephens 2020, 2022b).

The silos of higher education have perpetuated climate isolationism by emphasizing and supporting physical science and technological innovation to address climate change. Despite efforts to diversify science and engineering, persistent racial, gendered, and economic injustices of our economy and educational systems perpetuate exclusive access to science and engineering (Valantine and Collins 2015). The lack of diversity within the fields of physical science and engineering limits the scope of inquiry and constrains the types of connections that are made among science, technology, and society (Stephens 2020). Many colleges and universities sustain patriarchal leadership structures as they promote technocratic individualistic goals prioritizing the future financial success of their students and alumni and partnerships that serve corporate interests. The financialization of higher education has limited institutional commitments to prioritizing the public good and civic engagement. The problematic influence of the private sector and corporate interests in higher education is clear when one considers how and why fossil fuel companies have strategically supported higher education research since the 1950s (Westervelt 2021). The influence of the Koch family on higher education research (Leonard 2019) is the most widely recognized higher education funding source to promote climate denial and strategically resist climate action, but a larger network of climate change counter movement (CCCM) has focused providing financial support to colleges and universities to strategically resist climate action and efforts to reduce fossil fuel reliance (McKie 2021; Westervelt 2021). Because higher education has been prioritizing private interests rather than the public good by accepting funds to amplify climate denial and promoting climate isolationism, transformational changes in how higher education functions and interacts with corporate interests, the public sector, and marginalized communities are needed.

While technology is an essential part of a transition toward a more just, equitable, and climate stable future, investments in physical science and technological innovation have not yet been adequately balanced with investments in social science, social infrastructure, social innovations, and social justice (Overland and Sovacool 2020). This lack of investment has failed to strengthen social ties, thereby reducing community resilience—our ability to collectively cope with and recover from crises (Aldrich 2012). The lack of investment in social innovation and social justice has also constrained our imaginations about the role and potential impact of higher education in society. This narrow approach has begun to shift as there is growing recognition that addressing climate change will require investing in transformative social, institutional, financial, and political changes (Overland and Sovacool 2020). Still, data on research grants and funding shows that higher education continues to emphasize natural science and technology-based climate research rather than climate social sciences (Overland and Sovacool 2020). If social science research and social innovation were prioritized and funded at a higher level, the technological innovation would be coupled more effectively with research to accelerate the accompanying social change. As influential innovative institutions, higher education has an opportunity to lead by example and change the discourse from a climate isolationist approach to a more holistic and integrated climate justice commitment across campus functions and initiatives.

Moving beyond climate isolationism in higher education also requires recognizing that colleges and universities shape the communities in which they are located. Providing good jobs and economic vitality is often an assumed role of higher education institutions in their local communities, however “town-gown” interactions and relationships are often contentious, particularly when the college or university seems to be extracting from rather than contributing to the local community (Mtawa and Wangenge-Ouma 2022). As community engaged research and experiential learning are increasingly encouraged in higher education, colleges and universities are reckoning directly with the potential for exploitative and extractive relationships with local communities (Riccio et al. 2022). In the USA, the public good of higher education is recognized through its tax-exempt status, which has become increasingly controversial because local municipalities are disadvantaged by the lower tax base (Baldwin 2021b). Some universities end up contributing more economic benefits to the private sector than they do to local communities (Baldwin 2021b; Quigley 2018). A PILOT (payment in lieu of taxes) assessment program, where universities voluntarily contribute to local municipalities, has been established in some places to make up for the loss in tax revenue. PILOT is designed to facilitate a direct economic contribution from the university to the local community; however, many universities contribute much less than the amount recommended in the PILOT assessment (Quigley 2018). Expanding direct investments by higher education toward funding public infrastructure used by both the campus and surrounding community could simultaneously advance multiple goals including climate justice goals. Examples include higher education investing in fare-free public transit, upgrading local water infrastructure, building efficiency, installing community-based clean energy microgrids, and contributing to community resilience initiatives in anticipation of more frequent and intense climate disruptions.

### Climate justice and antiracist leadership in higher education

Embracing a paradigm shift through a climate justice commitment within higher education would represent an integrated way to demonstrate antiracist leadership and operationalize antiracist principles. Growing understanding since the 1960s that sources of local pollution inequitably distribute environmental burdens to communities of color and poorer communities has underscored the need for environmental policies to acknowledge, prevent, and compensate for the legacy of ongoing environmental injustice (Bullard and Johnson 2000; Kimmell et al. 2021). Racial and environmental injustice are intricately linked to climate injustice: a legacy of extractive colonialism has culminated in point sources of environmental pollutants, including from fossil fuel extraction and refinement, intentionally disproportionately located in and near communities of color (Johnson and Wilkinson 2021). These same communities are more vulnerable to the impacts of climate change despite having historically contributed least to greenhouse gas emissions (Yeampierre 2020). The pattern of marginalized communities facing higher exposure to climate hazards holds true across scales, recognition of which brought climate justice to the international climate policy agenda at COP6 in 2000 (Schlosberg and Collins 2014). Educational transformation is an essential component of addressing the intersecting crises of racial injustice, economic precarity, and climate disruptions (Crow and Dabars 2015). If higher education institutions are committed to racial justice, they also need to commit themselves to climate justice: the two are inextricably linked.

There is growing concern that higher education may be exacerbating rather than ameliorating the interconnected crises of our time, by failing to advance pro-democratic civil discourse and reduce racial inequities and gender disparities (Abreu 2020; Crow 2008; Fitzpatrick 2019; Gardner et al. 2021; Hooks 2003). Within the USA, higher education has been upholding racism and white supremacy by claiming “racelessness” and failing to critically reflect on the role of structural racism in the establishment and functioning of knowledge production processes (Patton 2016). Many scholars have argued that rather than exemplifying diverse, inclusive enlightenment, higher education remains a colonized space where upholding scholarship with European origins and economic aims is prioritized and institutional resources are geared toward building corporate and military capacity without consideration for social repercussions (Grande 2008; Kelley 2018; paperson 2017; Patel 2021). Many institutions of higher educations are constantly undergoing physical territorial expansion. The growth of land-grant universities in the USA exemplifies the role of higher education in expropriation and violent colonization of indigenous lands; the federal government gave indigenous land to universities igniting a process of profit and capital accumulation (Lomawaima et al. 2021; paperson 2017). The capital accumulated by expropriated land may otherwise be considered stolen from the communities from whom that land was taken; a theft compounded by the continued failure of higher education to fulfill their PILOT contribution commitment to local communities (Ahtone and Lee 2021; Fernandez 2016).

The inadequacy of how most universities are responding to both racism and the climate crisis demonstrates how higher education is reinforcing rather than disrupting the concentration of wealth and power (including white supremacy and the influence of fossil fuel interests). To advance a just societal transition away from worsening climate suffering, the dominant knowledge paradigms on which current societal systems were built must be challenged (Ojha et al. 2022). Recent efforts by US colleges and universities to establish courses and curriculum centering diversity, equity, and inclusion demonstrate how these efforts are largely oriented toward the experiences of white people (Abrica et al. 2021; Gonzales et al. 2021). Despite claimed commitments to diversity, these do not challenge existing power structures or approaches to academic knowledge production that maintain systemic racism, instead neutralizing the concept without effecting change (Patton 2016). In parallel, while fossil fuel divestment has been adopted in some colleges and universities (Stephens et al. 2018), higher education generally continues to perpetuate reliance on fossil fuels in their own campus energy systems, endowments, and the retirement investments of their faculty and staff (Healy and Debski 2017; McLaughlin and Pell 2022; Stephens et al. 2017).

### Distinguishing climate justice action from climate action

The inadequacy of the last decade of climate action suggests that a more holistic approach is essential for transformation. Climate justice relates to both the root causes and impacts of climate change as they uphold or limit human rights and justice (Robinson and Shine 2018). By centering justice in efforts to address the climate crisis, climate justice expands far beyond the climate isolationist approach of simple greenhouse gas emissions reductions and decarbonization. Rather, it emphasizes actions to address the underlying causes and extent to which vulnerable frontline communities face disproportionate impacts of climate change and fossil fuel pollution, aspiring to “fair treatment of all people and [seeking] to rectify the environmental burdens posed by discriminatory policies and systems, and by climate change” (Grady-Benson and Sarathy 2015). Climate justice helps to frame understanding of the benefits and burdens of greenhouse gas mitigation, responsibility and capacity to respond to climate change, and how to minimize the burden of adaptation on the world’s most vulnerable (Jenkins 2018).

Climate justice includes several dimensions of justice; procedural and distributional justice are most frequently considered. Procedural justice refers to participation and engagement in decision making. Distributive or distributional justice focuses on the distribution of or access to impacts/burdens and resources/responses across groups (Hurlbert 2011). Climate justice can also include recognitional justice, understanding, and fairly representing difference in culture and perspectives (Martin et al. 2016)—and intergenerational justice—ensuring the protection of nature for and limiting risk pushed on future generations (Newell et al. 2021). Reparational justice, sometimes described as restorative justice, is a component of climate justice that seeks to redress past injustices and prevent future injustices (Táíwò 2022).

Climate justice is also aligned with and synergistic with energy justice. Energy justice can be considered an integral subset of climate justice that typically focuses on reducing disparities and inequities in energy system outcomes and processes at a local or regional grid level (McCauley and Heffron 2018). Energy justice addresses many of the same tensions between social and environmental dynamics of transformation as climate justice with a focus on the power dynamics within energy systems. Energy justice emphasizes the need to address and prevent harms that result from unevenly distributed burdens and benefits of the ways energy is generated and distributed (Baker 2021). Although energy justice is specifically concerned with energy resource flows, the environmental and health impacts of these flows closely link energy justice to environmental justice. Energy justice scholars recognize the reproduction of extractive colonial dynamics in the inequitable placement of pollution burdens and energy costs on marginalized, colonized, and racialized people (De Onis 2021). With increasing awareness of energy injustices and how many climate and energy policies are exacerbating inequities by disproportionately benefiting wealthy individuals and communities, policy attention has shifted recently to expand energy investments in low-income households (Bednar and Reames 2020; Jenkins et al. 2020; Reames 2016; Si and Stephens 2021).

Climate justice also requires large and integrated investment in climate adaptation (Barrett 2013; Weinrub and Giancatarino 2015). To reduce suffering for the most vulnerable households and communities, large direct investments are needed in building resilience including food access, clean water, and education in frontline communities. Climate justice requires “radical shifts in how we (a) build and sustain relationships, (b) manage uncertainty, disruption, grief, and shock, and (c) redistribute wealth, opportunity, risk, and accountability” (Roberts-Gregory 2021). To prioritize innovative climate justice initiatives that move beyond climate isolation, higher education must diversify and value integration of other kinds of expertise, experiences, and perspectives.

Another distinguishing factor is that climate justice integrates labor justice. Fair compensation for dignified work and the economic empowerment that accompanies it are essential components of climate justice. To be clear, fair pay alone is unlikely to overcome inequities based in structural legacies of centuries of—at best—discriminatory exclusion from economic and professional opportunity and economic predation. However, higher education cannot claim to have achieved climate justice or be working toward a just transition without essential components of climate justice including healthy working conditions, fair, livable pay, and benefits for all people working on campus (Ayele 2020; Gunn-Wright and Hockett 2019). A climate just future necessarily will feature different modalities and dynamics of labor than those associated with the extractive past (Bouzarovski 2022). People of color, women, and people from other marginalized groups are more likely to earn poverty-level wages, a vicious cycle that reduces future opportunities for economic security and harms mental and physical health (Cooper 2018; Pillay-van Wyk and Bradshaw 2017). People who are not impoverished are less vulnerable to the impacts of climate change (IPCC 2022). Many institutions of higher education outsource work such as in food service to contractors, which allows the institution to claim that all employees are paid above a living wage and benefits floor despite data showing that contract workers are paid below the living wage without health benefits or the same protections as employees—a tension that was particularly pronounced during the COVID-19 pandemic when workers including food service staff and janitors at some universities were laid off without pay (Burke 2020).

### Disrupting financial assumptions and structures

Investing in climate justice in higher education requires disrupting mainstream financial assumptions about higher education and how colleges and universities are funded. For higher education to reclaim its focus on the common good and restructure to prioritize the public good, public funding has to increase. Underfunding of higher education and of sustainability incentives both limit the capacity of higher education to implement climate justice (Eichhorn et al. 2022). The paradigm shift we are proposing requires a renewed commitment to public support for higher education, so that colleges and universities are no longer beholden to wealthy students, alumni, and donors and corporate interests.

Navigating the challenges and the opportunities for higher education to advance climate justice requires collective resourcefulness, innovative teaching and learning, networked strategizing, and a compelling vision for the future. To leverage the potential of higher education, institutional creativity and resourcefulness will be needed among those within institutions of higher education and also among political leaders and education advocates outside of colleges and universities. Higher education leaders and advocates need to reinvent and reimagine a role that does not require continuing to profit from and reinforce current systems. For example, universities have begun to prioritize the influx of money from private entities, turning into so-called real estate companies and modern-day company towns (Baldwin 2021a). Fossil fuel companies have been strategically funding college and university research for decades to advance research to protect their industry (Brulle and Dunlap 2021; Healy and Debski 2017; Pang 2021).

A collective resistance to the financialization and privatization of higher education is critical (Washburn 2005). With the pattern in recent decades of devastating reductions in public support for higher education, colleges and universities have become increasingly reliant on the private sector and philanthropic donations. Like many other aspects of society, higher education has become increasingly “financialized” in the USA and other parts of the world (Sörlin 2007; Stephens et al. 2008). The financialization of higher education has contributed to increased economic inequities; from exorbitant tuition prices that result in huge student debt burdens to universities’ contracting out staff for food services, custodial services, parking services, and other facilities management (Banerji 2018), universities have privatized many basic services. By prioritizing climate justice, higher education leaders would have a framework to resist the privatization and corporatization of higher education. Rather than further privatization, climate justice principles justify major increases in public funding for higher education for public benefit. This proposed paradigm shift requires simultaneous efforts within and outside higher education to align public investments with a renewed commitment to advancing climate justice as a public good.

## Applying climate justice frameworks to assess transformative opportunities in higher education

**Fig. 2:**
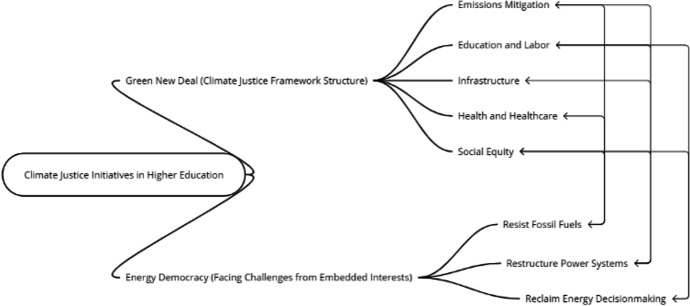
Conceptualizing climate justice frameworks for higher education. Applying two climate justice policy frameworks for higher education: (1) GND-based structure composed of five key interlinking topical areas, and (2) an Energy Democracy approach focuses on realigning power imbalances

This section provides a review of specific actionable initiatives and opportunities within higher education that we have identified that are aligned with transformative climate justice. To present the broad range of different kinds of initiatives that are aligned with advancing climate justice, we use two climate justice policy frameworks. Because a commitment to climate justice in higher education is complex, interconnected, and broad, these initiatives do not fit easily into traditional ways of thinking about the role of higher education in society. But by applying two climate justice policy frameworks that are not generally linked to higher education, we provide a novel approach to structure consideration of specific opportunities and initiatives for advancing climate justice in higher education. The two policy frameworks that align the processes and objectives of climate justice are the Green New Deal (GND) and GND-type policies and energy democracy (Fig. 2). GND-type policies link large public investments in clean energy with investments in labor, health, and education to strengthen community sustainability and resilience, building justice beyond more narrow conventional climate policies (Fig. 1) (Boyle et al. 2021). Energy democracy focuses on the main driver of climate change—dependence on fossil fuel energy and the power dynamics that underlie it—as a movement to resist fossil fuels, reclaim energy decision-making, and restructure energy systems for more equitable and renewable power (Burke and Stephens 2017).

The GND policy framework has inspired climate justice policies at all levels of governance—Indigenous, municipal, state, national, and regional—in many parts of the world, including in Europe, Canada, and South Korea (Boyle et al. 2021). Beyond the highly politicized Green New Deal proposal at the Federal level in the USA introduced in 2019, GND-like policies have emerged as climate justice policies because they acknowledge that climate change is an urgent crisis demanding rapid, transformative public investments that prioritize the advancement of social justice, racial justice, and economic justice rather than continuing to exacerbate social and economic disparities (Dalzell 2021). GND-type policies represent a new generation of climate policy focused on multi-systemic, transformational change that, crucially, centers social equity (Boyle et al. 2021). GND-type policies involve simultaneous large public investment in the future, including in renewable energy, public health, and just job creation and training (Fig. 1). In Table 1, we outline and describe these topical areas addressed in GND-type policies and how they apply to higher education.

**Table 1:** Applying the Green New Deal policy framework to higher education

Topical area	Description	Application to higher education
Climate mitigation	Includes: • renewable energy standards and • emissions goals for sectors	Set renewable energy and emissions reductions goals for university buildings, transportation, and heat and power
Education, labor, and workforce training	Includes: • education and retraining for a just transition, • green job development, and • labor rights	• Prioritize creating good jobs for workers from under-invested-in communities • Pay all employees a living wage with competitive benefits – end subcontracting lower-paid workers • Provide all employees with training to do their jobs effectively and sustainably • Incorporate sustainability and equity into curricula
Infrastructure investment	Framed as: • creating jobs or • spurring emissions reductions through clean energy	Invest in renewable energy and retrofit projects for the institution that provide good jobs to community members and may serve as learning and/or research opportunities for students and faculty
Health outcomes & healthcare	References: • negative impacts of climate change on human health • the harms experienced by frontline communities • deficiencies in the healthcare system exposed by COVID-19	• Identify the university’s role in community health (as a medical school or benefit-providing employer) • Ensure that all members of the university community and neighborhood have access to adequate and equitably delivered healthcare • Actively prevent or mitigate health harms such as from exposure to air pollution or other environmental toxins
Social equity	Addressed by mechanisms including: • reparations • empowerment in local investment decision-making • focused investment in a just transition for frontline communities • prioritized allocation of infrastructure investment and clean energy benefits to disadvantaged communities • procurement and contracting that prioritizes underrepresented communities	• Prioritize involvement, feedback, and where appropriate, leadership from representatives of under-invested-in communities including neighborhood community leaders • Co-design and co-develop projects and commit to equitable procurement and investment for infrastructure, research, hiring, and enrollment

Energy democracy embraces the idea that transformation away from centralized fossil fuel-based energy systems to a distributed renewable-based future can also redistribute power—political and economic power as well as electric power (Burke 2018; Feldpausch-Parker et al. 2022; Stephens 2019). Three kinds of innovative activities are central to the energy democracy movement: (1) resist centralized and concentrated fossil fuel based power, (2) reclaim decision-making in energy systems for the public good, and (3) restructure to encourage and support distributed renewable power (Burke and Stephens 2017). A key feature of energy democracy is the recognition that “how” renewable energy is deployed—that is, who is included, who is excluded, and how the benefits are distributed—matters a lot. Similarly to Table 1, in Table 2, we describe the three elements of energy democracy and how each applies to higher education.

**Table 2:** Applying the energy democracy framework to higher education

Energy democracy activity	Application to higher education
Resisting the legacy energy agenda that continues to support fossil fuels	Resist carbon-intensive energy: • divesting endowment from fossil fuels • refusing fossil fuel industry research funding • restricting fossil fuel industry representation on the board
Reclaiming energy decision-making so that the public interest is prioritized over corporate interests	Reclaim the energy system through genuine partnership with neighboring communities to develop community renewable energy options that meet the needs of the community and the university
Restructuring energy systems to maximize distributed local and regional benefits	Restructure the university’s unique role as a hub of innovation to co-design and co-produce • local renewable energy projects • microgrids • virtual power plants • demand management programs • other experimental clean energy projects

The climate justice policy frameworks of both the GND and energy democracy provide a valuable structure for considering the broad range of institutional initiatives that could advance climate justice in higher education.

## Novel university initiatives aligned with transformative climate justice

This section reviews a broad range of proposed university initiatives that would align with an institutional commitment to advancing climate justice. These proposed initiatives are based on a review of the literature on connections between the core elements of each climate justice policy framework and institutions of higher education. We detail specific potential climate justice initiatives first within the GND policy framework (Table 3) and then within the energy democracy framework (Table 4).

**Table 3:** Potential higher education climate justice initiatives within the GND framework

Topical area	Motivations	Actions in higher education
Climate mitigation	Fossil fuels are the core cause of climate change, and climate justice necessitates cessation of these activities (Newell and Simms 2020; Piggot et al. 2020; Strauch et al. 2020; Welsby et al. 2021)	End fossil fuel reliance in energy, transportation, food systems, and infrastructure: • emissions standards for new buildings and inputs thereto (Röck et al. 2020; Eichhorn et al. 2022) • retrofit old buildings for efficiency (Shen et al. 2019; Hao et al. 2020; Akkose et al. 2021; Rodrigues and Freire 2021) • eliminate fossil fuel inputs in campus landscaping and maintenance (Habib and Al-Ghamdi 2020) • limiting water and other resource consumption (Poland and Dooris 2010) • invest in renewable energy production and storage (Mathiesen et al. 2011; IPCC 2018) • use solely electric vehicles and promoting walking, cycling, etc. (Lutsey 2015) • limit air travel for presentations and conferences (Eichhorn et al. 2022; Kelly et al. 2022a) • initiate public advocacy efforts for climate policy by university government affairs offices and supporting academics in climate advocacy efforts (Kelly et al. 2022a, b)
Education, labor, and workforce training	A shift in curriculum and funding toward sustainability, climate justice and social justice education will be a large part of universities’ contribution to a greener future that includes economic justice (Muttitt and Kartha 2020). Unionization provides crucial protection of worker rights and is a worker right in itself. The concept of a just transition originates in the labor movement, in recognition of the need for good, green, healthy livelihoods to replace fossil fuel-driven jobs (McCauley and Heffron 2018). People who are healthy and not impoverished are more resilient and able to adapt to climate change and manage other hardships (Newell and Mulvaney 2013; D'Alessandro et al. 2020; Omann et al. 2020). Implementing climate justice requires not only addressing historic injustices, but eliminating ongoing and preventing future inequities and vulnerabilities (Sovacool et al. 2019)	Job Training: • offer transdisciplinary classes that pertain to high-skilled green jobs, from renewable energy engineering to sustainability policy • teach civic engagement and advocacy • build partnerships to develop training and re-training programs for a broader range of green jobs across skill levels – available at lower costs to more people (Trencher et al. 2017; Hoinle et al. 2021) • University of Vermont, for example, created a sustainability requirement—all students must take a general education course involving sustainability (The University of Vermont 2021) • engage with diverse pedagogies including Indigenous knowledge and the arts (Kelly et al. 2022b) Labor: • pay all employees a livable wage for the area’s cost of living • provide fair benefits • hire employees directly rather than sub-contracting to avoid paying living wages and benefits • allow (or encourage) unionization of all kinds of staff (McCauley and Heffron 2018)
Infrastructure investment	Physically increasing green space provides a benefit to the community in and around the university (McFarland et al. 2008). Renewable energy and reduced impact consumption of all kinds is possible on campuses. Plants are not only beneficial to the aesthetic quality of universities and mental health of the community members; they mitigate climate change by moderating the heat island effects and air pollution at the local level and by sequestering carbon (Edmondson et al. 2016; Willis and Petrokofsky 2017)	Invest in renewable energy and retrofit projects that provide community-accessible good jobs and learning and/or research opportunities • utilize campus rooftops as green spaces and building “living buildings” (Alfieri et al, 2009; Whittinghill and Rowe 2012; Köhler and Kaiser 2021) • provide resources to transport people to natural spaces outside of campus to increase the benefits of student engagement with the natural environment (Hartig et al. 1991; Benfield et al. 2015)
Health outcomes & healthcare	Universities can prioritize health equity in student and employee healthcare, which addresses both health injustice and environmental health injustices more specifically (Poland and Dooris 2010; Braveman et al. 2011). Community member health is also driven by access to sustainable and nutritious food. Universities can contribute to changing food access patterns and reducing food apartheid nearby (Brones 2018)	• Monitor and remediate issues in environmental health indicators like air and water quality in tandem with sustainability upgrades, such as through a weatherization plus health initiative that increases building energy efficiency (Underhill 2018; Tonn et al. 2021) • Address health injustices in the wider community by opening health services to the public (e.g. free, walk-in, or mobile health clinics) or offering price reductions in services for community members (Yu et al. 2017) • Work with farmers and local food distributors to make the university food system more sustainable by supporting local, sustainable producers on campus (Stahlbrand 2018) • Offer free or reduced-price meal plans at a fair sliding scale would address food inequities (El Zein et al. 2019) • Fund urban farms and gardens with local leaders that can function as educational tools as well • Apply behavioral psychology such as defaulting to vegetarian options in cafeterias and for catered events and placing vegetarian menu items at the top of menus. This can be highly effective in reducing carbon-intensive food consumption in cafeterias (Gravert and Kurz 2021)
Social equity	Universities have a history of gentrifying (or “studentifying” (Anderson 2015)) the areas around them, pushing out low-income residents of color, an injustice that must be acknowledged and addressed if committed to social justice. Higher education is often physically sited on land stolen from indigenous peoples; this land is considered capital that has accumulated for the institution at the expense of the original guardians of the land (paperson 2017; Lomawaima et al. 2021)	• Support resilient neighboring communities and engage to avoid gentrification • Acknowledge and making reparations to Indigenous tribes whose land was stolen to develop universities, and address local needs with university capital (Garton 2020; Lee and Ahtone 2020) • Invest in mixed-use buildings, affordable housing within off-campus buildings, and most importantly, listening to the surrounding neighborhoods as part of a full community engagement initiative before siting a new project (Evans et al. 2007; Garton 2020; Kopp 2021) • Build and maintain strong, restorative relationships with local communities and indigenous tribes on whose land a university is situated (Johnson et al, 2016)

**Table 4:** Higher education climate justice initiatives within the energy democracy framework

Action type	Motivations	Actions in higher education
Resist	Resistance includes efforts to delegitimize the fossil fuel industry, to reduce the political influence of fossil fuel interests, and to halt investments in fossil fuel infrastructure that are perpetuating fossil fuel reliance • Universities hold large endowments in the form of mixed investment portfolios. These investments are often directly or indirectly in the fossil fuel sector, such that the university is literally betting that the fossil fuel and its subsidiary industries will continue to prosper (through resource extraction and the life-threatening externalities it produces including climate change, air pollution, and water pollution) and is directly supporting it in doing so	• Fossil fuel divestment of institutional financial portfolios • Fossil fuel divestment of employee retirement account • End activities associated with expansion of fossil fuel production including divesting from fossil fuel-based companies such as the plastics and industrial agriculture industries • Refuse research and educational funding from fossil fuel interests, and restrict fossil fuel interests in university governance including board of trustees
Reclaim	Information transparency may include providing straightforward access to organized environmental data. Accessible data on sustainability efforts, achievement, and planning is vital for informed decision-making (Ferrer-Balas et al. 2010) • Mechanisms to hold the administration accountable are limited, and the board of trustees often self-perpetuates a cycle of member election, unclear criteria for board member conduct, and lack of meaningful opportunities for governance by student and faculty governing boards (Thelin 2003; Lubash 2015). Students, faculty, and staff also lack legal standing to hold universities accountable for achieving their stated mission • Corporations that make substantial donations to the university have a say in decision-making and strategy, given corporate ties to the board of directors (Barringer and Riffe 2018; Scott 2018). Trustees often lack academic experience as educators and staff, instead coming from corporate backgrounds with profit maximization mindsets (Queeney 2021)	• STARS reporting • Social justice data sharing like the Just program • Share more accessible financial statements • Empower the community to participate in decision-making discussions • Create clear behavior standards for members of university governance, in particular associations with fossil fuel and other extractive, unsustainable industries
Restructure	Increase public funding for higher education so institutions not reliant on corporate support and influence Socially responsible investing, particularly as part of divestment from fossil fuels, is a legitimate financial strategy that may produce returns at a higher rate over the long term than unethical or non-socially responsible investing (Sanzillo et al. 2018). The fossil fuel industry is continually weakening, losing its place at the forefront of effective investment strategy	Reinvest in green energy, social and economic justice, and community development • Ensure 100% renewable energy on campus and beyond • Invest divested funds in just and community-based enterprises

### Investing in transformation: initiatives compatible with the GND framework

**Fig. 3:**
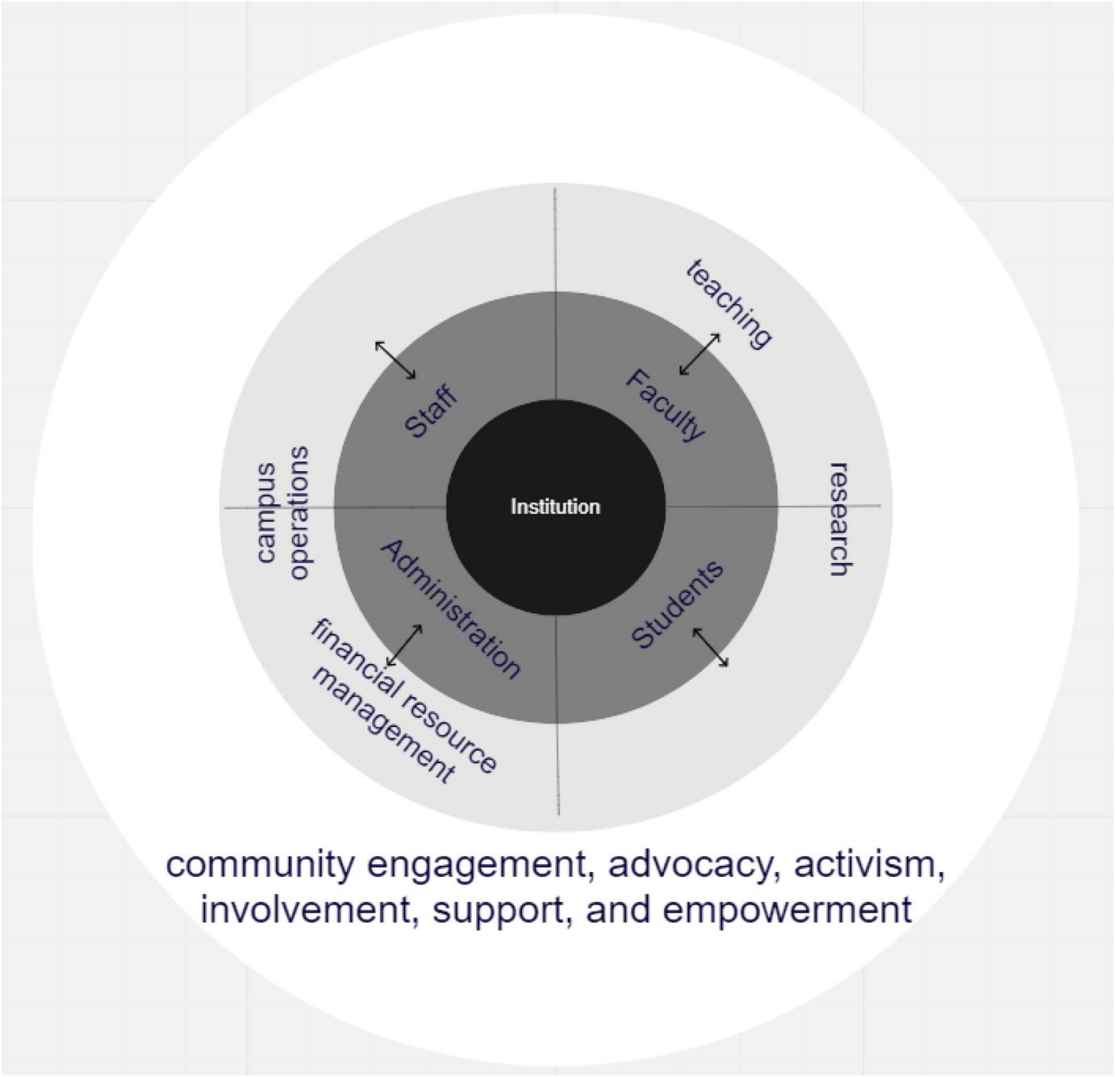
Higher education roles in climate justice. Internally, colleges and universities are composed of four primary constituents: Administration, Staff, Faculty, and Students/Alumni. These groups collectively engage in and are responsible for initiatives and programs in which climate justice (see Tables 3 and 4) can be embedded

In Table 3, we detail specific potential initiatives GND-like policies based on literature review of actions that institutions of higher education could incorporate in a climate justice plan. Several organizations, including the University and College Union in the UK and collegiate hubs of the Sunrise Movement, have begun organizing to support the implementation of GND principles in higher education; these efforts have included declaring a climate emergency and practicing ethical investment, in higher education: a GND for Universities (University and College Union 2021). These efforts lay the foundation for expanding the conception of the higher education sector as a key player in advancing social justice and transformative social change. The work of these organizations provides resources for colleges and universities so they do not need to develop an institutional climate justice plan from scratch. Building on the Sunrise Movement’s work, Table 3 provides innovative examples of climate justice initiatives in the five GND topical areas: (1) climate mitigation, (2) education, labor, and workforce training, (3) infrastructure investment, (4) health outcomes and healthcare, and (5) social equity. Initiatives in these areas can be integrated in and aligned with higher education’s basic functions of teaching, research, campus operations, financial resource management, and community engagement (Fig. 3) (McCowan 2020). The capacity of higher education community members to meaningfully engage with and connect to the broader community is cross-cutting; all groups’ activities influence and are influenced by social and policy change (Fig. 3). Given that most higher education institutions, whether public or private, are supported by both public and private funding, a substantial increase in public funding is required to reduce the impact and reliance on corporate interests and private sector actors whose priorities may not be aligned with climate justice goals. Among those advocating for transformative increases in public funding for higher education, Scholars for a New Deal in Higher Education emphasize how generous government funding and fair, inclusive governance of higher education is a necessary foundation for a democratic, equitable, and just society (SFNDHE 2022).

### Rebalancing power: initiatives compatible with the energy democracy framework

While the GND framework provides a structure to consider the potential of climate justice initiatives by linking climate and energy with jobs, health, infrastructure and equity, the energy democracy framework provides a conceptual structure to consider how climate justice initiatives challenge existing power dynamics (Fig. 2, Table 4). Vested interests benefit from the status quo, so the biggest challenge to implementing climate justice in higher education is managing fallout from disruptions to business as usual. Fossil fuel divestment, for example, has been contentious on many campuses because powerful forces do not want to lose profit from the continuation of a fossil fuel-based economy (Stephens et al. 2018). Resistance to fossil fuel interests, a key dimension of the energy democracy framework, is increasingly acknowledged as essential to combatting climate change and advancing climate justice (Newell et al. 2021). Resisting the dominant legacy of fossil-fuel-based energy systems includes resisting processes, technologies, and institutional and cultural norms (Newell et al. 2022; Stephens 2019). Given how fossil fuel interests have been strategically investing in higher education for decades as part of their effort to deny and delay transformative climate action, resisting fossil fuel interests in colleges and universities is an essential part of climate justice. Recognizing how challenging it is to shift power dynamics in higher education, this section explores potential climate justice implementation in higher education by discussing each component of energy democracy—resist, reclaim, and restructure.

#### Resist

Resisting fossil fuel interests in higher education is a critically important part of a commitment to climate justice (UnKoch My Campus 2020). Colleges and universities have been an important focus in the growing social movement to divest institutional endowments from fossil fuels (Stephens et al. 2018). A variety of educational organizations, philanthropic foundations, faith-based organizations, public pension funds, and non-governmental organizations have committed to divestment, and thousands of individuals have removed fossil fuel companies from their retirement and investment accounts (Stephens et al. 2018). Divestment is a tactic to target the fossil fuel industry through institutional investors, a key pillar of support upholding the industry’s political and economic dominance (Grady-Benson and Sarathy 2015). Divestment in US higher education derives from a student group at Swarthmore College in Pennsylvania—Swarthmore Mountain Justice—launching the first campaign for fossil fuel divestment in 2011 (Grady-Benson and Sarathy 2015). In September 2021, Harvard University, the most-endowed university in the world, divested from fossil fuels (Treisman 2021). Harvard had already eliminated direct investments in fossil fuels, so its new pledge will end the indirect investments that make up 2% of its ($42 billion) endowment. While divestment plays a role in delegitimizing the fossil fuel industry, divestment alone does not address fossil fuel connections in research funding and employee retirement funds.

Resisting the influence of fossil fuel investments in research and teaching in higher education is connected to transformative climate justice in many ways including prioritizing the fundamental knowledge creation and knowledge dissemination roles of higher education. With expanding research highlighting how strategic research funding by fossil fuel interests has been promoting misinformation and public ignorance rather than knowledge dissemination (Supran et al. 2023; Franta and Supran 2017; Oreskes 2019), a commitment to climate justice requires higher education leaders to resist fossil fuel interests. Growing research on agnotology, the intentional creation of ignorance, shows how powerful industries strategically deploy misinformation to create ignorance, confusion, and uncertainty such as by funding research that aligns with their priorities and suppressing research that does not (Corneliussen 2014; Hess 2020; Lewandowsky et al. 2015). These tactics are particularly relevant to climate research and advocacy, where the fossil fuel industry has funded climate science denial to delay environmental and climate action and suppress regulatory support for environmental social movements for decades (Hess and Belletto 2022; Oreskes and Conway 2011; Proctor 2010).

#### Reclaim

Another challenge is the lack of transparency and community-driven governance in most higher education institutions. The power and influence of faculty governance in higher education has been in decline, as senior administrators increasingly make strategic decisions without the input of faculty or students (Fitzpatrick 2019; Vican et al. 2020). Reclaiming decision-making to build climate justice institutions and infrastructure requires inclusive governance processes, including community accountability for leaders. Universities have an opportunity to innovate and demonstrate how to create just processes for decision-making. To implement meaningful justice and equity into a climate justice policy, universities should strive for transparent and inclusive governance systems (UnKoch My Campus 2020). Climate justice policies in higher education could take many forms with social justice at the core of planning and investments. Policy creation must incorporate procedural justice, meaning all stakeholders—not only wealthy investors—have a say. Successful climate justice requires changes in practice with tangible benefits.

#### Restructure

In addition to resisting corporate interests including fossil fuels and reclaiming decision-making, a restructuring of higher education incentives, financing, and purpose is needed to reorient the mission toward the public good and climate justice. Restructuring the way research projects and campus energy infrastructure projects are designed and conducted, to include co-design and co-development with non-academic communities, creates opportunities for building local relationships and prosperity. For example, universities as anchor institutions can link more closely their intellectual and practical innovations with external communities and partners (Brown and Bozuwa 2017). Higher education could partner with the Fossil Fuel Non-Proliferation Treaty initiative, a network of hundreds of civil society organizations with thousands of academic signatories, formed in recognition of the importance of committing to phasing out fossil fuels and reinvesting in a just transition beyond international commitments such as under the Paris Agreement to advance research, learning, and innovation toward this goal (Newell et al. 2022; Treaty 2022).

Restructuring higher education toward advancing societal goals of transformative climate justice clearly requires change and innovation at multiple levels including changes beyond what can be achieved at any individual college or university. Broader innovations in educational policy and public funding for higher education are essential for the paradigm shift that we are exploring here. There is a growing movement calling for a new scale of transformative public investment for higher education, i.e., Scholars for New Deal for Higher Education (SFNDHE 2022).

## Conclusions

As the climate crisis continues to exacerbate vulnerabilities around the world, a paradigm shift in how higher education engages with transformative social change could alter policy options for a more just and climate stable future. Higher education is a critical sector of society that is so-far under-leveraged in terms of preparing for the future.

This conceptual contribution suggests that unless and until climate justice is prioritized and embraced within higher education, colleges and universities are not only sustaining the status quo, but are further reinforcing climate vulnerabilities and exacerbating and perpetuating climate injustices.

Universities are critically important places of knowledge production, knowledge perpetuation, and knowledge dissemination, so during this era of climate disruption and instability they can apply this knowledge capacity to advance systemic social change (Stephens et al. 2008). By leveraging their resources, including their intellectual resources, their human capital, and their physical resources, higher education institutions can become both exemplars of social change and agents for change. As anchor institutions, colleges and universities are among the most stable and forward-looking organizations in many communities (Sladek 2019). To reimagine what is possible, the climate crisis must be addressed within higher education in conjunction with systemic change for social justice and structural change focused on economic justice, racial justice, and energy justice.

A commitment to climate justice provides an intersectoral and interdisciplinary approach to identifying research priorities, training students, interacting with local governments and communities, and supporting employees. Campus operations including renewable energy generation and procuring equipment and food provide other opportunities for demonstrating innovations. This opportunity for leadership in reprioritizing educational and research goals toward transformative structural change for climate justice is amplified among institutions with large endowments who have more financial flexibility in demonstrating and catalyzing change.

To achieve their missions of advancing the common good through learning and innovation, colleges and universities can leverage their resources and reprioritize and reorient their educational and research initiatives toward building transformational climate justice in society. New kinds of strategic collaborative relationships and partnerships with non-academic partners at multiple scales are critical to expanding beyond traditional siloed academic work. New kinds of collaborations may include co-producing climate justice knowledge with community partnerships, expanding international, global research partnerships particularly among interdisciplinary teams in the global north and global south, and growing intergenerational research such as early career and graduate training opportunities, and cross-cutting curricula linking climate justice across disciplines, programs, departments, and schools.

Prioritizing climate justice is an opportunity for colleges and universities to commit to leading the way in ending fossil fuel reliance including shifting how they invest endowments and eliminating unethical petrochemical funding sources. To realize this potential, higher education institutions need to resist corporate influence and recommit to advancing the cutting edge of research and practice for the public good.

Many universities—even those that have made ambitious climate commitments—have not yet leveraged the opportunity to lead the transformation toward a climate just society. Courage and a commitment to advancing social justice are necessary for universities to overcome the many challenges of innovating for climate justice (Bartlett 2021). Climate justice requires a community-engaged approach that expands beyond the individual institution of higher education. A paradigm shift in how higher education is funded is also a key part of enabling and empowering colleges and universities to prioritize climate justice and embrace a transformative lens to better address the interconnected crises of our time.

## Data Availability

The authors confirm that the data supporting the findings of this study are available within the article and its supplementary materials.
